# MicroRNA hsa-mir-3923 serves as a diagnostic and prognostic biomarker for gastric carcinoma

**DOI:** 10.1038/s41598-020-61633-8

**Published:** 2020-03-13

**Authors:** Xiaohui Yang, Ze Zhang, Lichao Zhang, Li Zhou

**Affiliations:** 1 Department of Obstetrics & Gynecology, The First Hospital of Jilin University, Changchun, Jilin 130021 China; 2 Department of General Surgery, The First Hospital of Jilin University, Changchun, Jilin 130021 China; 3 Department of Parasitology of Zhongshan School of Medicine, Sun Yat-sen University, Guangzhou, 510080 China

**Keywords:** Tumour biomarkers, Risk factors

## Abstract

Gastric carcinoma (GC) refers to a common digestive system disease that exhibits a very high incidence. MicroRNA hsa-mir-3923 belongs to a type of miRNA, of which the function has been merely investigated in breast, pancreatic cancers and pre-neoplasic stages of gastric cancer. It has not been studied or reported in gastric carcinoma, so the relationship between gastric hsa-mir-3923 expression and the clinics feature and pathology of GC cases was examined. This study employed data mining for analyzing gastric carcinoma data in The Cancer Genome Atlas database. A Chi squared test was performed for assessing the relations of hsa-mir-3923 expression with clinics-related and pathology-regulated variables. This study conducted the assessment of the role of hsa-mir-3923 in prognostic process using Kaplan–Meier curves, Receiver operating characteristic (ROC) analysis and proportional hazards model (Cox) study. With the use of Gene Expression Omnibus, this study carried out gene set enrichment analysis (GSEA). In the meantime, the common miRNA database was compared to predict potential target genes; as revealed by co-expression analysis, a regulatory network probably existed, containing hsa-mir-3923. For the analysis of the most tightly associated cytological behavior and pathway in GC, this study adopted the databases for Annotation, Visualization and Integrated Discovery (David) and KO-Based Annotation System (KOBAS). Cytoscape, R and STRING were employed for mapping probable regulatory networks displaying relations to hsa-mir-3923. Lastly, we obtained 69 genes most tightly associated with hsa-mir-3923 and described their relationship with Circos plot. As revealed from the results, hsa-mir-3923 displayed up-regulation in gastric carcinoma, and it displayed associations with vital status, N stage and histologic grade when being expressed. The predicted results of miRNA target genes suggested that there may be a close relationship between 66 genes and hsa-mir-3923 in gastric cancer. As indicated from co-expression data, a small regulating network of 4 genes probably existed. Our results elucidated that hsa-mir-3923 high-expression reveals poor prognosis of GC patients.

## Introduction

Gastric carcinoma (GC) refers to a type of common malignant tumor in digestive tract. It exhibits high mortality and poor prognosis^1^. Optimized treatment (e.g., effective surgery and neoadjuvant treatment) cannot enhance its overall survival (OS) rate^2^. Thus, novel biomarkers specific to GC should be identified for disease detection in early stage. This effort can be beneficial to start the early treatment of GC and enhance the prognosis of patients.

MicroRNA can noticeably affect the study on cancer in terms of tumor’s biological activity (e.g., tumor transcription, epigenetic regulation, as well as expression of gene)^3^. They are capable of interacting with mRNA or lncRNA, leading to the formation of pathway in human cancers^4^. As demonstrated by a number of examples, the interaction between lncRNA OIP5-AS1 and miR-186a can inhibit ZEB1 expression in hepatocellular carcinoma (HCC)^5^, thereby impairing the metastasis of tumor cell. MiRNA is also able to affect the apoptosis, autophagy, EMT and other cytological behaviors^6^. The present study focused on miRNA hsa-mir-3923, which is capable of regulating some diseases and some biological behaviors^2,7^. For instance, it is beneficial to invade and metastasize cancer lymph nodes^8^. According to the recent study, Dr Li identified that lncRNA-NUTF2P3-001 could inhibit the pathway of hsa-mir-3923/KRAS affected by oxidative stress^7^, which affected the proliferation of tumor; nevertheless, the role of hsa-mir-3923 as a specific marker in the gastric carcinoma should be explained.

This study aimed at identifying the pathological function exhibited by hsa-mir-3923 in GC. The study carried out a retrospective analysis on data originating from tissue chip data (GSE13195 & GSE30727) and The Cancer Genome Atlas Stomach Adenocarcinoma (TCGA-STAD) cohort. The assessment was conducted for the underlying prognostic implication exhibited by hsa-mir-3923 in GC patients, as well as the independent prognostic value it displays for the OS of GC patients. Subsequently, the Gene Set Enrichment Analysis (GSEA) helped to gain more insights into hsa-mir-3923 regulatory mechanism-related proteins and biological functions. The target gene of this type of special miRNA was searched in numerous common miRNA databases first, followed by drawing the comparison of these data with those achieved from TCGA and GEO. As suggested from the results, 66 genes were tightly associated with the hsa-mir-3923 in the gastric cancer tissues. Cytological pathway and behavior which displayed tight association in GC were studied based on KO-Based Annotation System (KOBAS) as well as Database for Annotation, Visualization and Integrated Discovery (DAVID), STRING. Subsequently, genes in mentioned databases were combined for screening mRNA, lncRNA as well as miRNA related to the hsa-mir-3923 in terms of expression. With the use of STRING, Cytoscape and R, the hsa-mir-3923 related regulatory networks were mapped. A set mount of genes showing the closest association with has-mir-3923 were harvested, and the relation between them and the Circos plot was described. While studying GC patients, how hsa-mir-3923 expression affects the patients regarding their clinical characteristics was measured. Survival curve analysis could be beneficial to determine its diagnostic performance, and Cox regression analysis was conducted for the assessment of the predictive effect on OS and the relapse-free survival. The paper also discussed the biological activities hsa-mir-3923 participated and possible effects hsa-mir-3923 imposed.

## Materials and Methods

### Data acquisition and collection

RTCGA Toolbox package (version 3.5) in R (version 3.5.3) provided the data of gastric carcinoma cases and RNA-seq expression outcomes^9,10^. Additionally, this study achieved the expression data of hsa-mir-3923 tumor from TCGA in terms of several digestive tumors, covering stomach, pancreas, liver, esophagus, colon and bile duct. The GEO database (https://www.ncbi.nlm.nih.gov/geo/) provided gene microarray with cancer tissue data (GSE13195 & GSE30727)^11^. In the mentioned databases in June 2019, this study obtained the data employed here^12^.

### Statistical analyses

SPSS software 23.0 (IBM Corporation, Armonk, NY, USA) was employed for data analyzing. This study adopted R/Bioconductor package of edgeR for determining miRNAs with differential expression based on TCGA STAD dataset^13^. All thresholds were set at the absolute log2(count + 1) fold change (tumor/normal) ≥ 2 and the false discovery rate (FDR) < 0.01. Boxplots were adopted in terms of discrete variables for the measurement of diversifications in expression, and influences exerted by clinicopathological characteristics on hsa-mir-3923 expression were studied by Kolmogorov-Smirnov test (K-S test). This study presented alterations in expression between respective group by scatter plots. χ^2^ tests were adopted for examining the correlation of hsa-mir-3923 expression and clinical data^14^. GraphPad Prism 7.0 software (GraphPad Software, Inc.) was employed for analyzing the differentially expressed condition of hsa-mir-3923 in a range of tumor tissues^15^. Scatter plots and histograms were adopted for discrete parameters for measuring diversifications in expression between a range of tissues, and influences exerted by tumor tissue of origin on hsa-mir-3923 expression were analyzed using the mean ± SD. Receiver-operating characteristic curve (ROC) was plotted by “p-ROC package” (version 1.0.3)^16^ for evaluating the diagnosing ability we divide cases to groups with high and low hsa-mir-3923 expression by the best cutoff value of OS determined by the Youden index^17^. Correlation coefficient analyses were performed using R software; a correlation coefficient R > 0.5 was taken into account for indicating a strong correlation^18^. Kaplan–Meier curves were adopted for the comparison of the diversifications in the overall survival and relapse-free survival using survival package in R^19^. Univariate Cox analysis was used to select the related variables. Subsequently, the Multivariate Cox analysis was employed on the effect exerted by hsa-mir-3923 expression on the overall survival and relapse-free survival of cases^16^.

### Gene set enrichment analysis (GSEA)

GSEA refers to a computational approach determining if an a priori defined set of genes is of statistical significance, concordant diversification between two biological states. Here, GSEA was carried out with the GSEA software 3.0 from the Broad Institute^20^. The gene expression data referred to RNA-seq data from GEO and TCGA-STAD database. The gene set of “c2. cp.biocarta.v6.2.symbols.gmt”, “c3.cp.biocarta.v6.2.symbols.gmt”, “c5.cp.biocarta.v6.2.symbols.gmt” and “h.all.v6.2.symbols.gmt”, summarizing and representing specific, well-defined biological states or processes, originated from the Molecular Signatures Database (http://software.broadinstitute.org/gsea/msigdb/index.jsp)^21^. The normalized enrichment score (NES) was calculated by analysis with permutations for 1,000 times. A gene set shows significant enrichment at a normal P-value of <0.05 and false discovery rate (FDR) of <0.25.

### Gene enrichment and functional annotation evaluation

The Database for Annotation, Visualization, and Integrated Discovery (DAVID; http://david.abcc.ncifcrf.gov/)^22^ & STRING (https://string-db.org/)^23^ & KO-Based Annotation System (KOBAS) (http://kobas.cbi.pku.edu.cn/)^24^ were adopted for conducting related pathway analysis^25^, and Kyoto Encyclopedia of Genes and Genomes (KEGG) pathway and Gene ontology (GO) term enrichment analysis was carried out for the functional annotation of the co-expressed genes^26^. Three GO terms [molecular function (MF), cellular component (CC) and biological process (BP)] were employed for identifying the enrichment of target genes. GO terms and KEGG pathways with P-values <0.05 were of statistical significance. With the use of Cytoscape, the enrichment map of annotation analysis was made (version 3.3.1) (http://www.cytoscape.org/cy 3.html)^27^.

### Prediction of related genes

Comparative analysis several miRNA databases, such as miRDB (http://www.mirdb.org/miRDB/), miRPathDB (https://mpd.bioinf.uni-sb.de/), TargetScan (http://www.targetscan.org/), miRNAWalk (http://zmf.umm.uni-heidelberg.de/apps/zmf/mirwalk2/), miRTarBase (http://mirtarbase.mbc.nctu.edu.tw/php/index) etc. for finding lncRNAs that have regulatory relationships with hsa-mir-3923 and mRNAs that this special miRNA may regulate^28^. Subsequently, Venn package (version 1.8)^29^ and UpSetR package (version 1.4.0)^30^ in R were used to draw Venn graph and UpSet plots in STAD, suggesting the interactions among the results of five miRNA databases. When the comparison of TCGA and GEO databases was drawn, the possible related genes in GC were estimated and then made in Cytoscape.

### Weighted co-expression network construction

The co-expression study module of WGCNA’s result (“WGCNA” package in R) can harvest genes co-expressed with hsa-mir-3923, to build a weighted correlation network by WGCNA^31^. For WGCNA, the R package DCGL (version 2.1.2) was adopted for filtering genes; we took genes with FPKM values >0.85 to conduct subsequent study. The adjacency matrix between a range of genes was built with 3 as the variable of soft thresholding power to decrease noise and false relation. In brief, weighted correlation matrices were transformed into matrices of connection strengths using a power function^32^. The mentioned link strengths were subsequently adopted for calculating topological overlap, a robust and biologically valuable measuring process which compacts the co-expressing correlation of three various genes in the network^33^. Note that among hsa-mir-3923 and three indirectly linked genes, the built matrix information is 96% overlapped with the gene prediction results from miRNA databases. To avoid repetition, the co-expression network was not built, or biological function enrichment analysis was not performed again. The related genes found by miRNA databases and data form GEO as well as TCGA belong to gain Circos plots. According to the re-mappings of two tables from the mentioned two tools, Circos plots was produced. With the use of the Circos visualization tool in R (version 3.5.3), Circos plots were made^34^.

### Genome map

When the gene expression matrixes of GC cases in three databases were integrated, the correlation coefficient between different genes and hsa-mir-3923 (calculation method reference statistic part) was calculated, and the Venn diagram with GraphPad on the calculated results was drawn to verify the feasibility of the results achieved in the co-expression study again. Alinements of gene co-expression maps to GRCh38.95 (reference genome version of Homo sapiens) (ftp://ftp.ncbi.nlm.nih.gov/genomes/all/GCA_000001405.15_GRCh38.95)^35^ found reference genomic regions promoting the composition of 69 genome sets (the location of the genes of interest in the human reference genome). To search for the most typical reference fragments, all GRCh38.95 loci presenting in the gene co-expressing maps were derived and integrated, so 69 reference donor fragments were achieved, as fixed in the outermost track. The number of gene regulation maps containing the tags of each fragment is then labeled in the second orbital, excluding the double count. In the internal layer, gene pairs showing regulatory relationships were linked. The sum of the genome map alignments across the entire genome was adopted to link the 69 gene regulatory maps in the Circos plot^36^.

## Results

### Patients’ characteristics

The Cancer Genome Atlas (TCGA) database provided both gene expression and clinical data of cases with gastric carcinoma. The overall number of cases was 452. When the first screening was achieved, 25 tumor samples and 3 normal samples were removed with excessive number of lost or ambiguous data, and the rest 39 normal samples and 385 tumor samples could be achieved. The specific clinical characteristics, covering ethnic compositions, pathological status, survival status, TNM stage, gender, and age are listed in Table 1.

**Table 1:** Demographic and clinical characteristics of TCGA-STAD cohort. (n = 385).

Characteristics	Number of sample size (%)	
Age (years)	No	Percentage
≤55	69	17.92%
>55	316	82.08%
**Gender**		
Female	145	37.66%
Male	240	62.34%
**T stage**		
T1	18	4.68%
T2	81	21.04%
T3	177	45.97%
T4	100	25.97%
Tx & Unknown	9	2.34%
**M stage**		
M0	344	89.35%
M1	27	7.01%
Mx & Unknown	14	3.64%
**N stage**		
N0	116	30.13%
N1	103	26.75%
N2	77	20.00%
N3	75	19.48%
Nx & Unknown	14	3.64%
**Clinical Stage**		
I	50	12.99%
II	115	29.87%
III	161	41.81%
IV	37	9.61%
Unknown	22	5.71%
**Histologic grade**		
G1	8	2.08%
G2	134	34.80%
G3	236	61.30%
Gx & Unknown	7	1.82%
**Vital status**		
Death	248	64.42%
Survival	137	35.58%
**Race**		
Asian	79	20.52%
Black	11	2.86%
White	245	63.64%
Unknown	50	12.99%
**Total**	385	100%

### MicroRNA hsa-mir-3923 high-expression in GC

**Figure 1:**
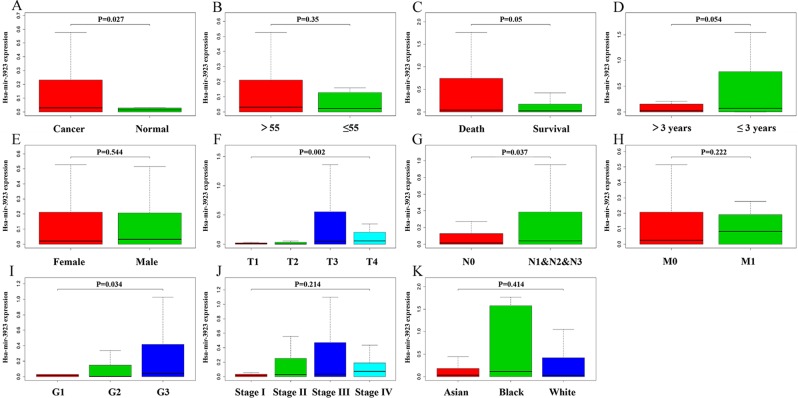
Boxplots showing differences in hsa-mir-3923 expression according to tissue type (**A**), patient age (**B**), vital status (**C**), survival time (**D**), gender (**E**), TNM stage(F&G&H), pathological status (**I**), clinical stage (**J**) and race (**K**).

**Figure 2:**
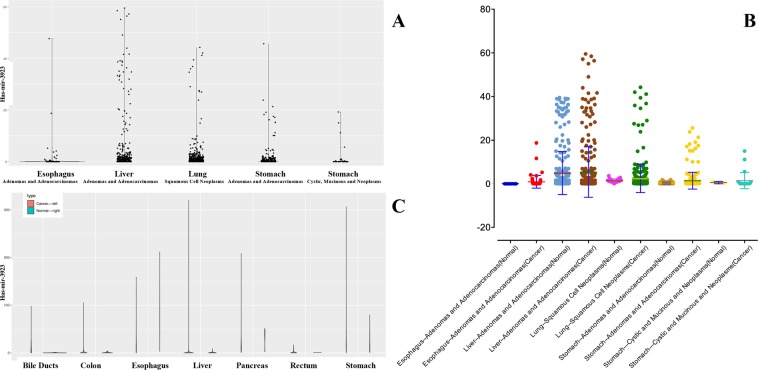
miRNA hsa-mir-3923 expression in several common tumors based on TCGA database. (**A**) illustrates the expression of this special miRNA in several common tumor tissues in the TCGA database. (**B,C**) comparing the expression of hsa-mir-3923 in tumor tissues and normal tissues, and the expression level of this special miRNA in the tumor tissues was significantly increased. The expression level of hsa-mir-3923 in tumors is higher than that of normal tissues. (Note: The miRNA hsa-mir-3923 was found recently, so very few clinical patient data will contain this special miRNA data. Only clinical samples containing this special miRNA with a large amount of data would be included in the calculation.).

With the use of boxplots, the diversification in hsa-mir-3923 expression in GC cases and normal people were ascertained. Figure 1 suggests that the overall expression trend of hsa-mir-3923 in GC was assessed, and subsequently hsa-mir-3923 expression was reported to be remarkably higher in primary cancer tissues than in normal gastric tissues (P = 0.027; Fig. 1A). Besides, distinct hsa-mir-3923 expressions existed in the groups by vital status (P = 0.050; Fig. 1D), T stage (P = 0.002; Fig. 1E), N stage (P = 0.037; Fig. 1F) and histologic grade (P = 0.034; Fig. 1I). It is noteworthy that diversifications in hsa-mir-3923 expression were identified in accordance with patient age as well as TNM stage, gender, race and other clinicopathological parameters (Fig. 1). hsa-mir-3923 expression data in several common tumors were also harvested from TCGA database (Fig. 2A). (Note: For the late discovery of this miRNA, there is no such miRNA expression data in numerous tumor types. The statistical analysis was only conducted on the tumor types with this special miRNA expression and large sample size.) After horizontal comparison, hsa-mir-3923 expression was reported to be up-regulated in tumors from a range of organs and tissues (Fig. 2B,C).

### The relationship between hsa-mir-3923 expression and clinical features in GC

In accordance with Chi-square tests, the relationship of the clinical features with the expression of hsa-mir-3923 was studied and enumerated in Table 2. hsa-mir-3923 expression displayed tight relations to vital status (X^2^ = 4.829, P = 0.028).

**Table 2:** Correlations of mirRNAhsa-mir-3923 expression in patients with STAD tissue with clinicopathologic variables.

ClinicalCharacteristics	Variables	No. of patients	miRNA hsa-mir-3923 expression		X^2^	P-value
			High No.	Low No.		
**Age(years)**	≤55	69	30	39	1.488	0.223
	å 55	316	163	153		
**Gender**	Female	145	69	76	0.602	0.438
	Male	240	124	116		
**Clinical stage**	Stage I/II	165	75	90	1.552	0.231
	Stage III/IV	198	103	95		
	Unknown	22	15	7		
**Grade**	G1/2	142	66	76	1.493	0.222
	G3/4	236	125	111		
	Gx (Not include)	7	2	5		
**T stage**	T1/2	99	43	56	1.957	0.162
	T3/4	277	143	134		
	Tx (Not included)	9	7	2		
**M stage**	M0	344	167	177	3.288	0.070
	M1	27	18	9		
	Mx (Not included)	14	6	8		
**N stage**	N0	116	50	66	2.614	0.106
	N1/2/3	255	133	122		
	Nx (Not included)	14	10	4		
**Vital Status**	Survival	248	114	134	**4.829**	**0.028**
	Death	137	79	58		
**Survival Time**	≤3 years	359	178	181	0.638	0.424
	å 3 years	26	15	11		
**Race**	White	245	118	127	0.598	0.439
	Asian	79	42	37		
	Black	11	6	5		
	Unknown	50	26	24		

Bold values of P ≤ 0.05 indicate statistically significant correlations.

### High hsa-mir-3923 expression as a single prognosis element for poor survival

**Figure 3:**
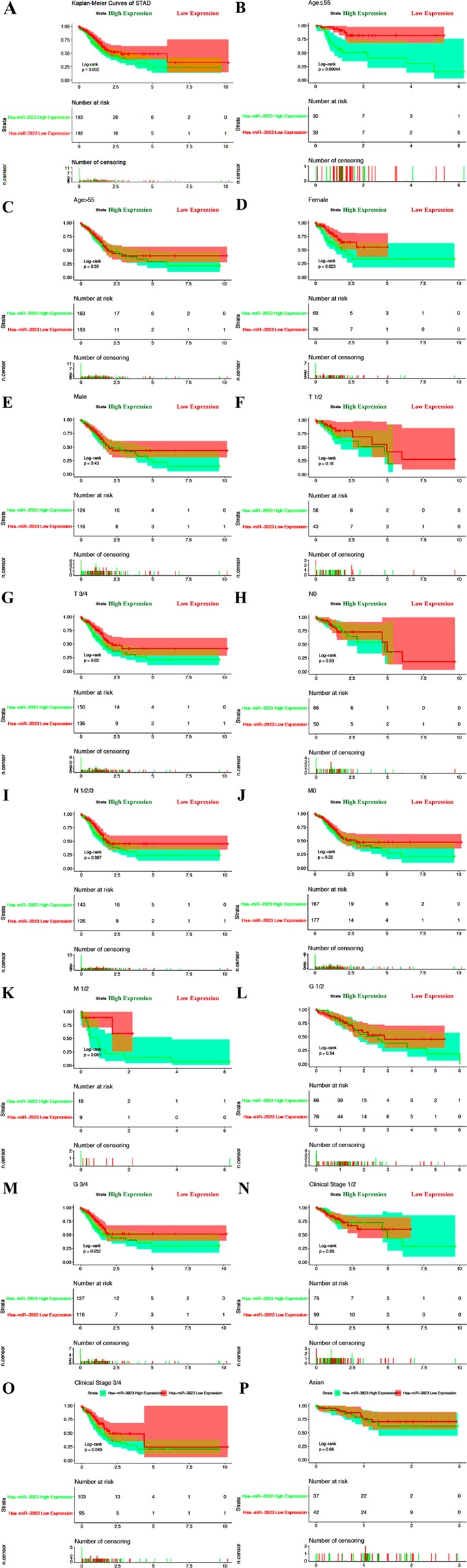
Kaplan-Meier curves for survival of STAD patients according to hsa-mir-3923 expression in GC tissues. Patients were divided into high and low hsa-mir-3923 expression groups using the Youden index of hsa-mir-3923 expression as the cut-point. Kaplan–Meier curves produced survival analysis (**A**) and subgroup analysis according to age (**B**,**C**), gender (**D**,**E**), TNM stage (From **F** to **K**), pathological status (**L**,**M**), clinical stage (**N**,**O**) and race (Asian) (**P**) were performed based on Kaplan-Meier curves. Note: For missing data, such as Tx, Nx, Mx, etc. In the statistical process, we referred to other information of the sample to classify the incomplete data into possible groups.

**Figure 4:**
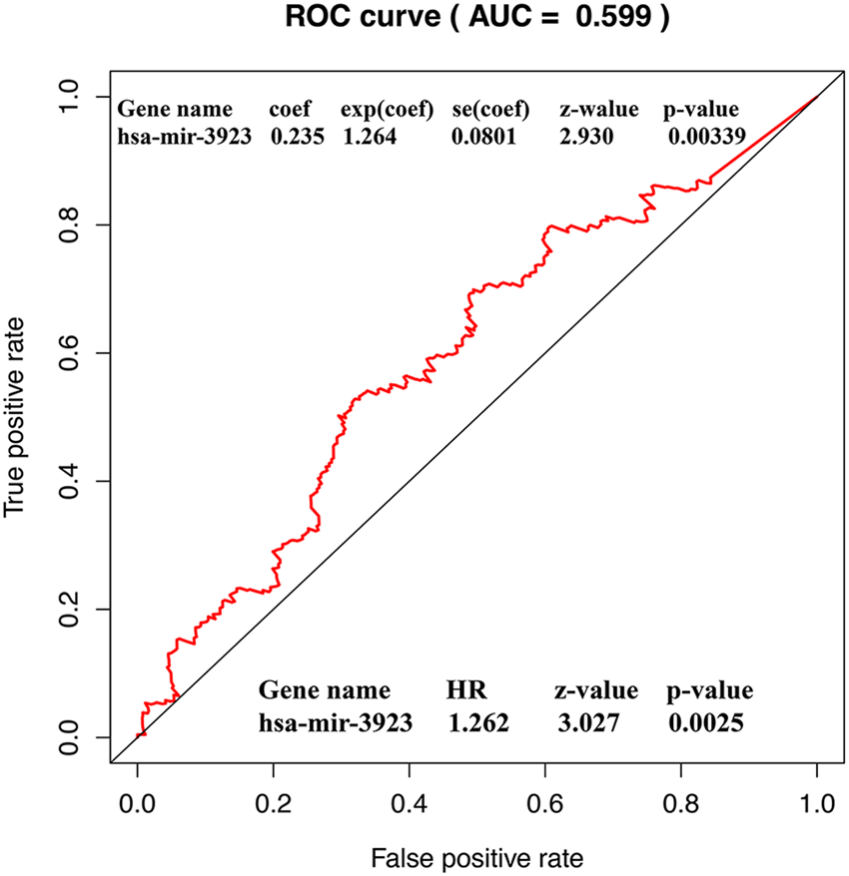
ROC curve to identify the optimal cutoff value for patients with GC. Abbreviations: AUC, area under the curve; ROC, receiver-operating characteristic curve.

Kaplan-Meier curves of overall survival were plotted, and log-rank tests revealed the relations of hsa-mir-3923 high-expression to poor overall survival (P = 0.032; Fig. 3A). As revealed from an in-depth subgroup analysis, hsa-mir-3923 high-expression displayed relations to poor overall survival of cases with age (P = 0.00044; Fig. 3B), gender (female) (P = 0.023; Fig. 3D), advanced T stage (T3/4) (P = 0.040; Fig. 3G), and advanced clinical stage (stage 3/4) (P = 0.049; Fig. 3O). Figure 4 shows that the area under the curve (AUC) was 0.599, representing the moderate diagnostic ability, and the ROC of hsa-mir-3923 was executed. In hsa-mir-3923 high-expression patients, we used univariate analysis selected the critical variables (e.g., TMN classification, pathological grade, clinical stage, gender and age). According to multivariate study with the Cox proportional hazards model, the expression of age (HR = 4.965, P = 0.026), gender (HR = 4.649, P = 0.032), clinical stage classification (HR = 1.796, P = 0.005) and T classification (HR = 2.165, P = 0.057) were single prognosis elements for GC cases (Table 3).

**Table 3:** Univariate and multivariate analyses of overall survival in high-expression of hsa-mir-3923 in patients with GC.

Parameters	Univariate Analysis			Multivariate Analysis		
	Hazard ratio	95% CI	P-value	Hazard ratio	95% CI	P-value
**Age, years (≤55 > 55)**	**4.965**	**1.003–1.052**	**0.026**			
**Gender (Female & Male)**	**4.649**	**0.356–0.952**	**0.031**			
**Race (Asian & Black & White)**	0.536	0.832–1.495	0.464	1.714	0.872–2.13	0.175
**Grade (G1 & G2 & G3 & G4)**	0.365	0.727–1.827	0.546	1.121	0.724–2.952	0.29
**Stage (Stage I & II & III & IV)**	1.478	0.769–2.501	0.078	**1.796**	**1.14–2.122**	**0.005**
**T stage (T1 & T2 & T3 & T4)**	1.843	0.707–2.525	0.191	2.165	1.269–3.120	0.057
**M stage (M0 & M1)**	0.016	0.31–3.795	0.898			
**N stage (N0 & N1/2/3)**	0.001	0.738–1.344	0.979			
**Hsa-mir-3923**	0.332	0.561–1.371	0.564			

Bold values indicate statistically significant, P <0.05.

### GSEA identifies the hsa-mir-3923 related biological functions and proteins

For the identification of biological functions excited in gastric carcinoma, data were screened out from tissue chips (GSE13195 & GSE30727) in the GEO database. The GSEA between high and low hsa-mir-3923 expression data sets was performed. GSEA reveals significant differences (FDR < 0.25, P-value<0.05) in the enrichment of “MSigDB Collection”, and Table 4 lists the specific contents. In the GC, hsa-mir-3923 participates in the anabolism of RNA in tumor cells, covering NLS (Nuclear localization sequence) bearing protein import into nucleus, piRNA metabolic process, regulation of alternative mRNA splicing via spliceosome, pre mRNA binding and enhancement of RNA polymerase activity, etc. Moreover, hsa-mir-3923 was reported probably participating in the sperm formation process, sperm maturation and the final fertilization process. Likewise, the results of KEGG pathways clarify that hsa-mir-3923 participates in the anabolism of RNA (e.g., RNA degradation and RNA polymerase). It may be wrapped in exosomes, probably acting as a marker for tumor detection. Furthermore, the miRNA can also be involved in the anabolism of proteins, and these signaling pathways cover glutamate metabolism and alanine aspartate, protein export, pyrimidine metabolism, cysteine and methionine metabolism, etc. Only the 20 most characteristic biological functions and signaling pathways were selected, as listed in Table 4.

**Table 4:** MicroRNA has-mir-3923 high-Expression related GO terms and KEGG pathways in GC.

GO Terms	Size	ES	NES	NOM p-value
GO_CYTOPLASMIC_STRESS_GRANULE	31	0.6298445	1.8949641	<0. 001
GO_CELLULAR_RESPONSE_TO_GAMMA_RADIATION	19	0.60617167	1.8890903	<0. 001
GO_NLS_BEARING_PROTEIN_IMPORT_INTO_NUCLEUS	21	0.72323453	1.8892685	0.00201207
GO_PIRNA_METABOLIC_PROCESS	15	0.6631818	1.6797496	0.00214592
GO_RNA_POLYMERASE_II_CARBOXY_TERMINAL_DOMAIN_KINASE_ACTIVITY	16	0.69689363	1.7967532	0.00795229
GO_POLY_PURINE_TRACT_BINDING	18	0.6448273	1.7354437	0.00804829
GO_REGULATION_OF_ALTERNATIVE_MRNA_SPLICING_VIA_SPLICEOSOME	34	0.5403653	1.690862	0.01022495
GO_TRANSCRIPTION_FROM_RNA_POLYMERASE_I_PROMOTER	34	0.579346	1.7645965	0.01174168
GO_FERTILIZATION	133	0.40344077	1.6190195	0.01174168
GO_SPERMATID_DIFFERENTIATION	109	0.36569467	1.5642152	0.01183432
GO_RNA_POLYADENYLATION	28	0.62098473	1.7526788	0.01221996
GO_NUCLEOTIDE_EXCISION_REPAIR_PREINCISION_COMPLEX_STABILIZATION	21	0.63953274	1.7727898	0.01271186
GO_CELLULAR_PROCESS_INVOLVED _IN_MULTICELLULAR_ORGANISM	223	0.3124185	1.4743642	0.01375246
GO_CARBOXY_TERMINAL_DOMAIN_PROTEIN_KINASE_COMPLEX	22	0.6530758	1.7933326	0.01596806
GO_HISTONE_MRNA_METABOLIC_PROCESS	24	0.6345253	1.7683904	0.01863354
GO_SPERM_EGG_RECOGNITION	43	0.5346265	1.6876888	0.01980198
GO_BINDING_OF_SPERM_TO_ZONA_PELLUCIDA	33	0.5493318	1.6699042	0.02330097
GO_SINGLE_FERTILIZATION	102	0.406942	1.5861306	0.02362205
GO_PRE_MRNA_BINDING	24	0.60193044	1.6801043	0.02464066
GO_CYTOPLASMIC_STRESS_GRANULE	125	0.43866456	1.6930021	0.02479339
GO_CELLULAR_RESPONSE_TO_GAMMA_RADIATION	31	0.6298445	1.8949641	<0. 001
GO_NLS_BEARING_PROTEIN_IMPORT_INTO_NUCLEUS	19	0.60617167	1.8890903	<0. 001
GO_PIRNA_METABOLIC_PROCESS	21	0.72323453	1.8892685	0.00201207

### Estimation of relevant genes and gene‐enrichment and functional annotation studies

**Figure 5:**
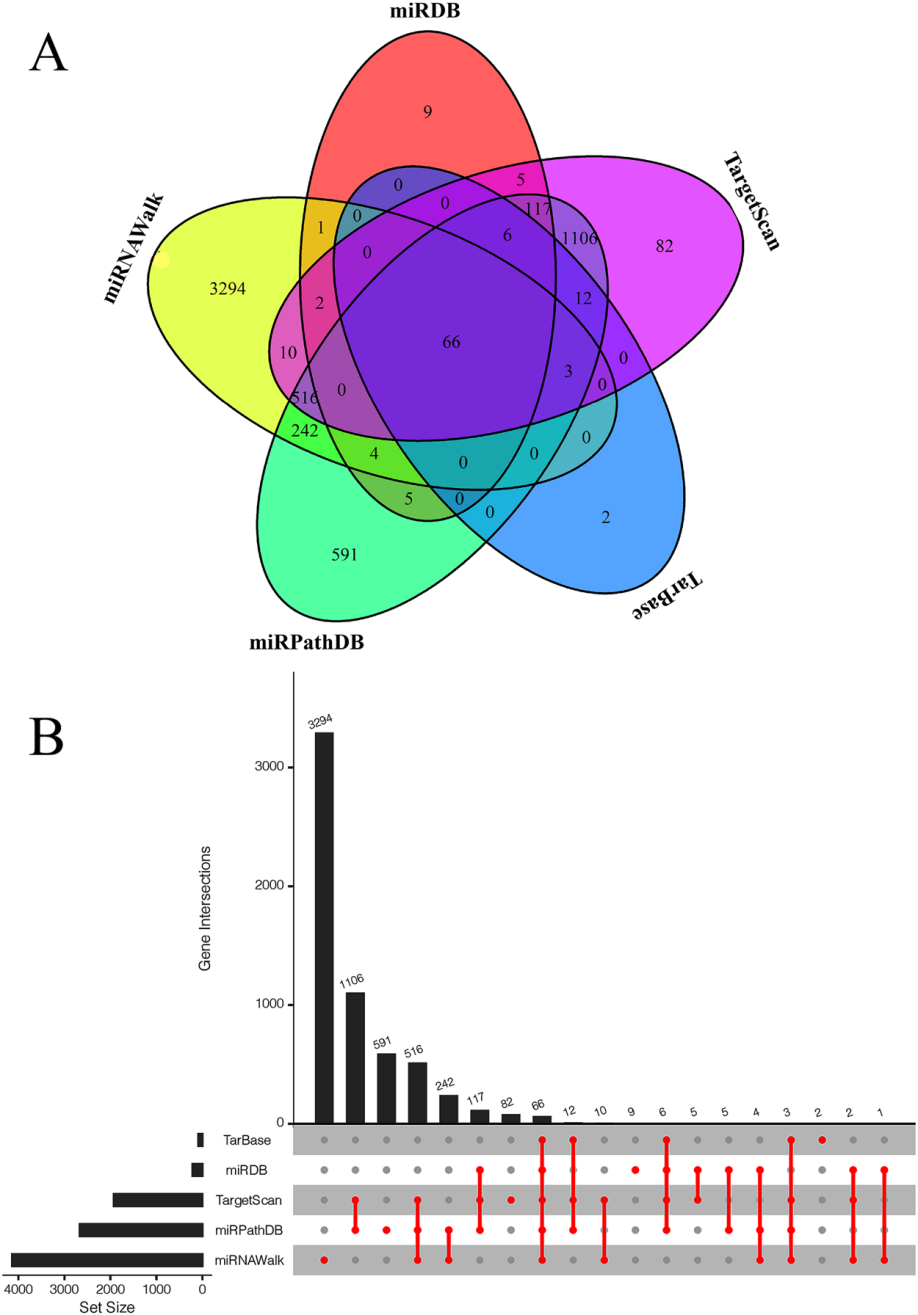
The potential target genes of hsa-mir-3923 are predicted in four common miRNA databases. The Venn diagram (**A**) and UpSet plot (**B**) showed that there might be 66 potential target genes of hsa-mir-3923. Please see Table 5 for details.

**Figure 6:**
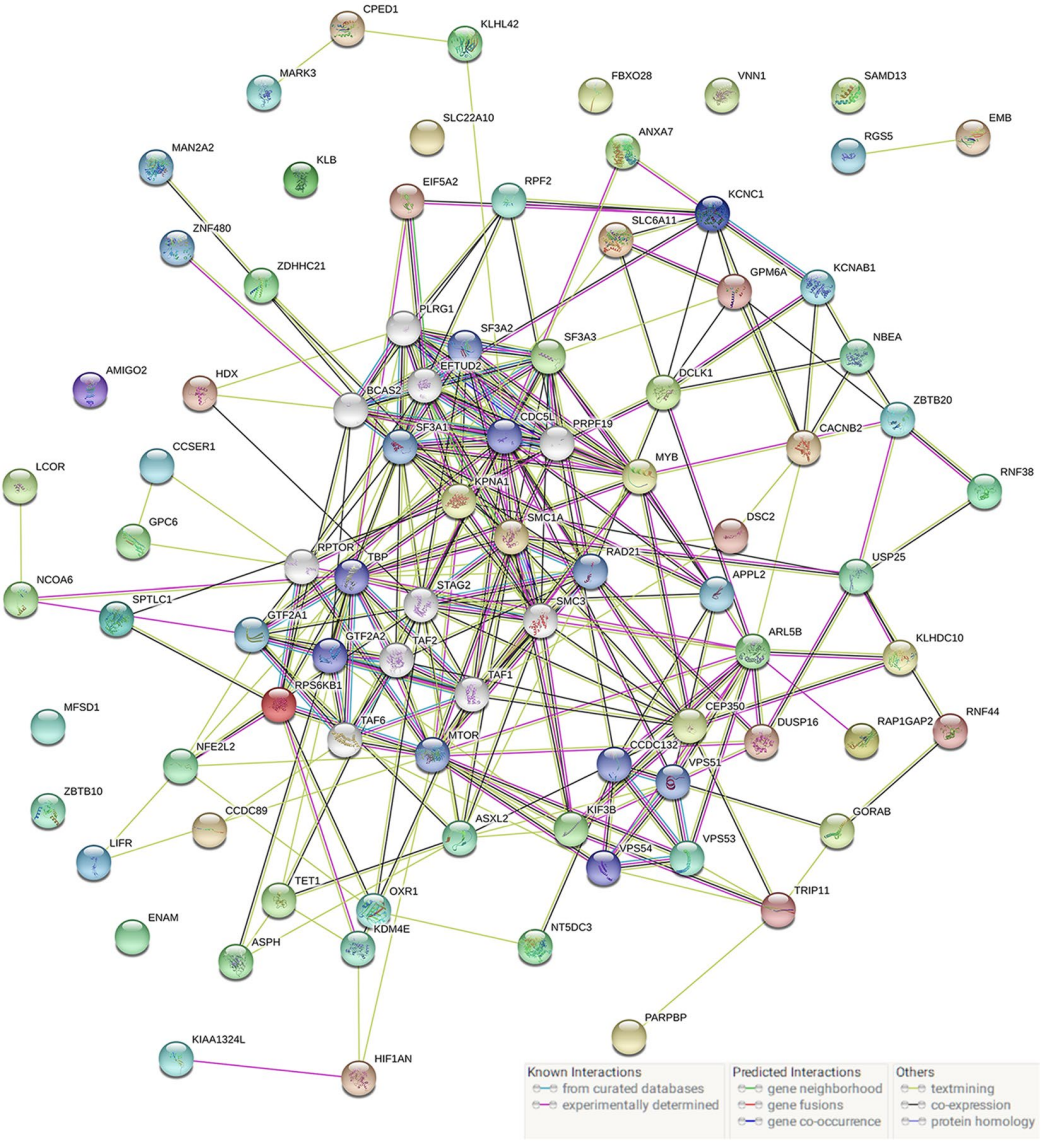
The functional protein association network enriched by STRING. The protein interaction network encoded by 66 target genes and has-mir-3923 is drawn with STRING. In this large network, there are many small regulatory networks, which have an impact on different biological functions.

**Figure 7:**
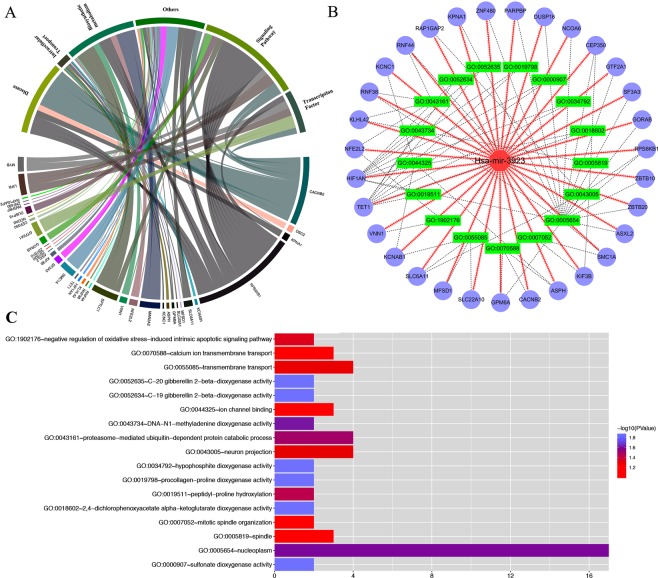
The significantly GO terms identified by DAVID and Cytoscape. R were used to conduct a visual enrichment analysis of genes enriched in the GO terms and to construct an interaction network for related genes. (**A**) shows that these target genes can affect these cytological behaviors. (**B**) shows the number of enrichments and correlations of target genes in various projects. (**C**) shows the number of enrichments of the target gene in each project. Abbreviation: GO, Gene Ontology; DAVID, Database for Annotation, Visualization and Integrated Discovery.

**Figure 8:**
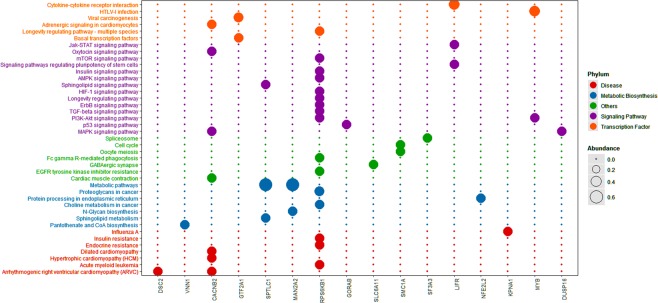
Significant KEGG pathway determined by KOBAS. The predicted 66 potential target genes can be enriched in 41 different KEGG pathways. These signaling pathways can be broadly divided into five categories, of which 16 genes and 3 signaling pathways are significantly correlated, as shown in Table 6. Abbreviation: KOBAS, KO-Based Annotation System; KEGG, Kyoto Encyclopedia of Genes and Genomes.

Several miRNA databases (e.g. miRDB, miRPathDB, TargetScan, miRNAWalk and miRTarBase) were comparatively analyzed to find lncRNAs with regulatory relationships with hsa-mir-3923 and mRNAs that this special miRNA may regulate. After the TCGA and GEO databases were compared, the probably relevant genes in GC were estimated. In Fig. 5, after comparing these five common miRNA databases, 66 more relevant target genes were identified to be probably present in gastric cancer tissues, each of which are presented in Table 5. First, STRING was used for enriching the functional protein relation network. By eliminating some nodes with no additional links, some small regulatory networks were found probably existing in the whole system, as shown in Fig. 6. By KOBAS and DAVID, we identified the noticeable GO terms and KEGG pathways. Cytoscape (version 3.3.1) was used for a visual enrichment study of genes up-regulated in the GO pathways and for building an interaction network for related genes (Fig. 7). Next, R was adopted to visually enrich KEGG pathways (Fig. 8) and these GO terms (Fig. 7). Table 6 clarifies that these genes are critical in the biological behaviors below: molecular function (MF) (2,4-dichlorophenoxyacetate alpha-ketoglutarate dioxygenase activity, hypophosphite dioxygenase activity, sulfonate dioxygenase activity, procollagen-proline dioxygenase activity, C-20 gibberellin 2-beta-dioxygenase activity, C-19 gibberellin 2-beta-dioxygenase activity, DNA-N1-methyladenine dioxygenase activity, ion channel binding), biological process (BP) (proteasome-mediated ubiquitin-dependent protein catabolic process, peptidyl-proline hydroxylation, negative regulation of oxidative stress-induced intrinsic apoptotic signaling pathway, transmembrane transport, calcium ion transmembrane transport, mitotic spindle organization) and cellular component (CC) (nucleoplasm, neuron projection, spindle). Besides the mentioned information regarding GO terms, hsa-mir-3923 is tightly associated with these KEGG pathways (e.g., pantothenate and CoA biosynthesis, MAPK signaling pathway) within GC cells, as well as the progression of arrhythmogenic right ventricular cardiomyopathy (ARVC).

**Table 5:** MicroRNA has-mir-3923 potential target gene and co-expression analysis prediction.

MicroRNA has-mir-3923 Target Gene Prediction					
Co-expression Network					
hsa-mir-3923		AC009646.2	OPRK1	AC117402.1	
**Target Gene**					
HDX	ARL5B	VPS53	DCLK1	MARK3	ZBTB20
SPTLC1	OXR1	RNF44	SF3A3	ANXA7	NFE2L2
CPED1	TET1	EIF5A2	KIF3B	ZNF480	DSC2
RNF38	TRIP11	ENAM	EMB	ASXL2	SMC1A
PARPBP	GPM6A	CCDC89	KCNC1	HIF1AN	CCSER1
CACNB2	DUSP16	KCNAB1	FBXO28	USP25	NCOA6
SLC22A10	AMIGO2	KLHL42	ASPH	ZBTB10	RAP1GAP2
GTF2A1	GPC6	ZDHHC21	SAMD13	RPS6KB1	NT5DC3
MFSD1	NBEA	KLB	CEP350	MAN2A2	APPL2
KPNA1	LIFR	SLC6A11	MYB	RGS5	KIAA1324L
RPF2	GORAB	LCOR	KLHDC10	KDM4E	VNN1

**Table 6:** GO terms and KEGG pathways of Hsa-mir-3923-related target genes involved.

GO ID	%	P-value	FDR	GO terms
GO:0018602	3.03030303	0.013908322	15.43396266	2,4-dichlorophenoxyacetate alpha-ketoglutarate dioxygenase activity
GO:0034792	3.03030303	0.013908322	15.43396266	hypophosphite dioxygenase activity
GO:0000907	3.03030303	0.013908322	15.43396266	sulfonate dioxygenase activity
GO:0019798	3.03030303	0.013908322	15.43396266	procollagen-proline dioxygenase activity
GO:0052635	3.03030303	0.013908322	15.43396266	C-20 gibberellin 2-beta-dioxygenase activity
GO:0052634	3.03030303	0.013908322	15.43396266	C-19 gibberellin 2-beta-dioxygenase activity
GO:0043734	3.03030303	0.024214599	25.42699877	DNA-N1-methyladenine dioxygenase activity
GO:0005654	25.75757576	0.024921803	24.84493411	nucleoplasm
GO:0043161	6.060606061	0.03161174	35.69479046	proteasome-mediated ubiquitin-dependent protein catabolic process
GO:0019511	3.03030303	0.036722635	40.20605433	peptidyl-proline hydroxylation
GO:1902176	3.03030303	0.046505801	48.03390393	negative regulation of oxidative stress-induced intrinsic apoptotic signaling pathway
GO:0043005	6.060606061	0.048789224	43.22517035	neuron projection
GO:0055085	6.060606061	0.049982177	50.57850615	transmembrane transport
**KEGG ID**	**R-Value**	**P-value**		**KEGG pathways**
hsa05412	0.615489462	0.012053686		Arrhythmogenic right ventricular cardiomyopathy (ARVC)
hsa00770	0.366615519	0.025140101		Pantothenate and CoA biosynthesis
hsa04010	0.312941482	0.036348782		MAPK signaling pathway

Abbreviation: GO: Gene Ontology. KEGG: Kyoto Encyclopedia of Genes and Genomes.

### Co-expression network construction

**Figure 9:**
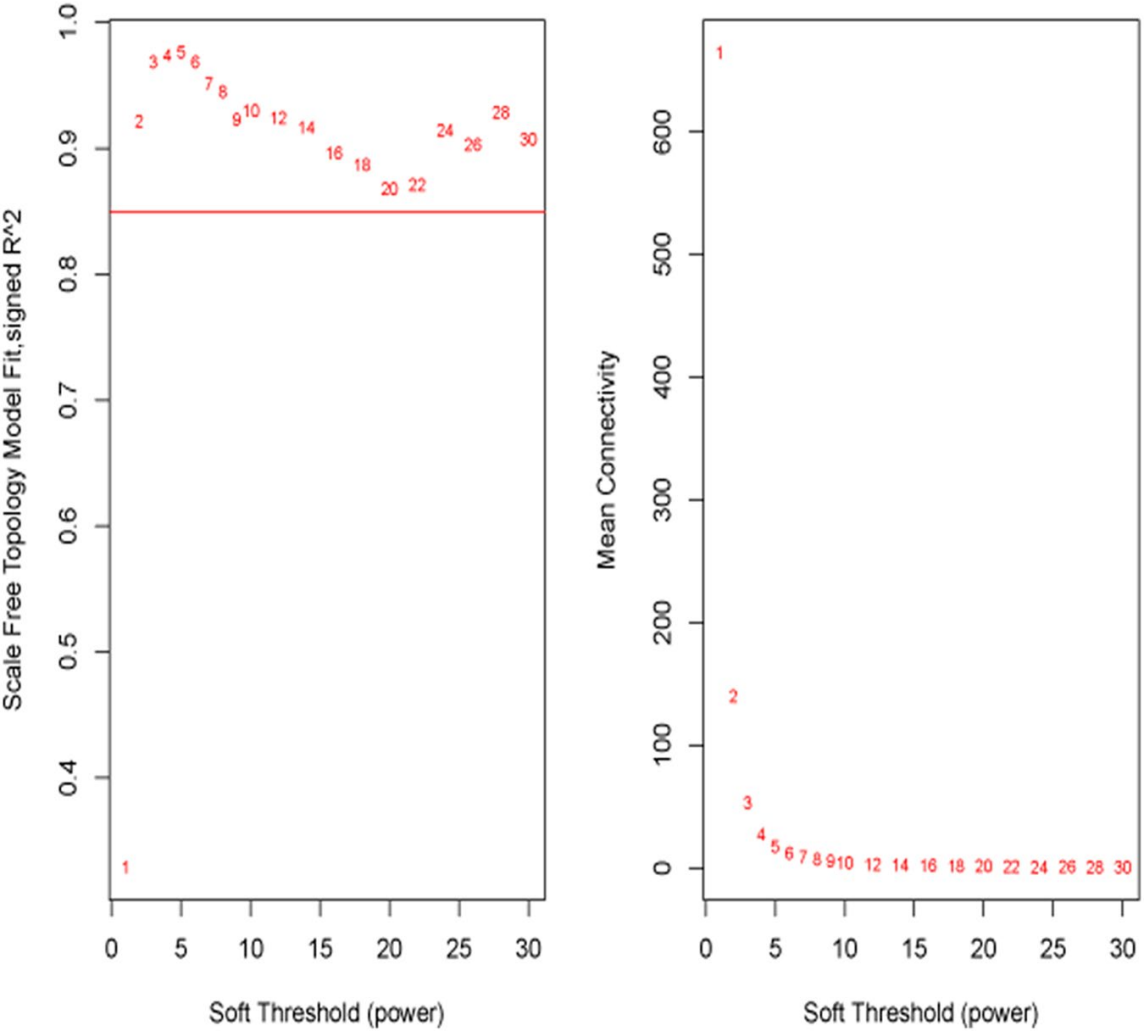
Determination of soft-thresholding power in the weighted gene co-expression network analysis (WGCNA). In the co-expression network, R is used to analyze the scale-free fitting index of various soft threshold powers (β) (Power Value = 0.85).

**Figure 10:**
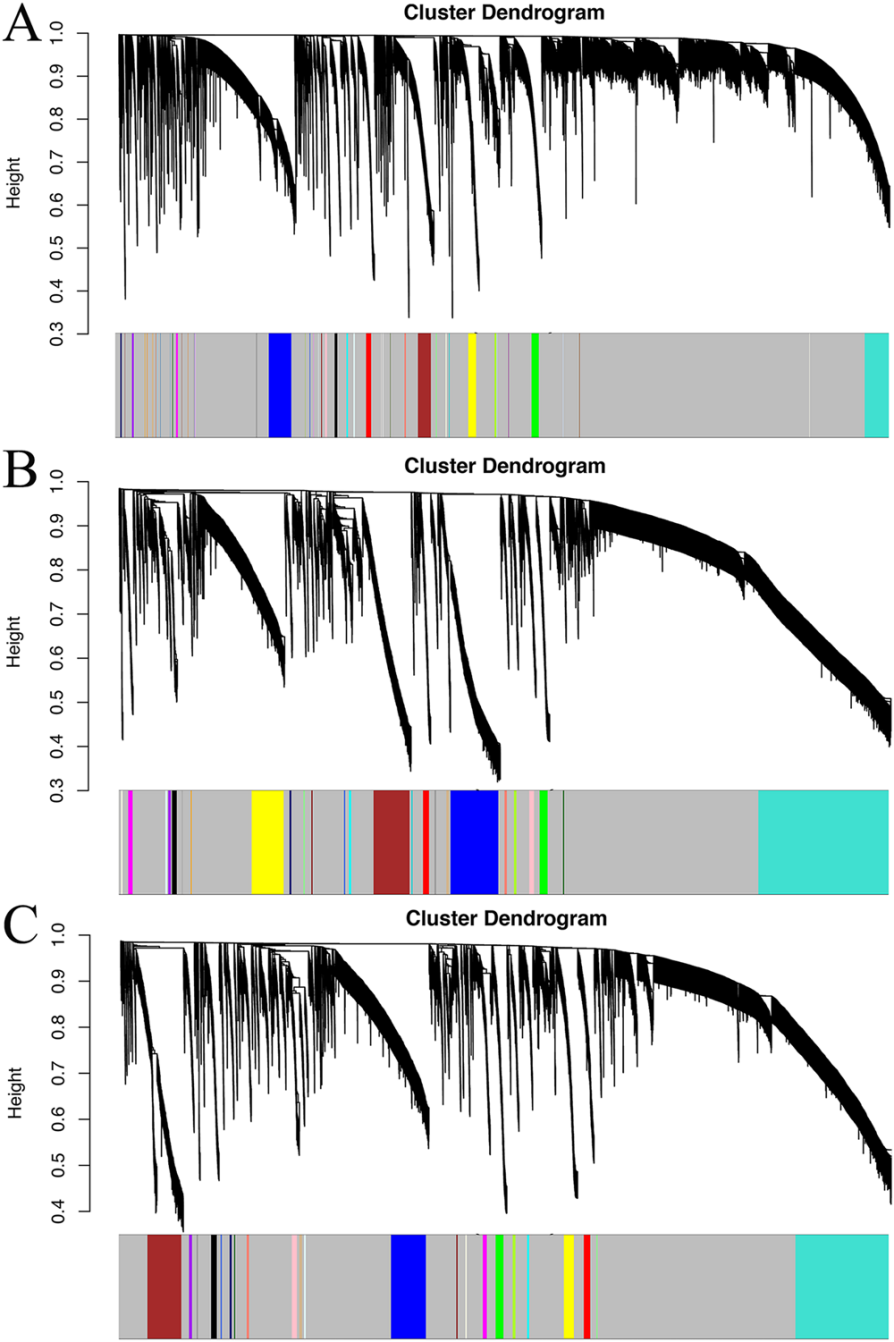
Visual Representations of the Gene Co-Expression Network. Hierarchical clustering of all differentially expressed genes and visualization of gene module partitioning. The colored bars (bottom) directly correspond to the different module (color) of the gene cluster. We eliminated the interfering genes by adjusting the screening criteria. (**A**) (log Fold Changeå 1, P-value <0.05), (**B**) (log Fold Changeå 2, P-value <0.05) and Fig. 11C (log Fold Changeå 2, P-value <0.01). In order to narrow the prediction range of the co-expression network, and to more accurately find the target gene with co-expression relationship with hsa-mir-3923.

First, R’s “edgr” package was used for calculating the diversification (log Fold Changeå 1, P-value <0.05) in expression among mRNA, miRNA and lncRNA in the TCGA-STAD and GEO (GSE13195 & GSE30727) database. Then, co-expression analysis on the data was performed again. WGCNA package in R was adopted for the analysis of the relationship between lncRNA, miRNA and mRNA (Power Value=0.85) (Fig. 9). Next, the gene associated with hsa-mir-3923 was taken for co-expression grid. Since the initial screening conditions are overly loose (Fig. 10A), the constructed co-expression network nodes are extremely sophisticated. Accordingly, the screening criteria were modified, and more rigorous screening conditions were exploited to build a more concise co-expression network. (log Fold Changeå 2, P-value <0.05) (Fig. 10B) & (log Fold Changeå 2, P-value <0.01) (Fig. 10C). Nevertheless, the result overlaps with over 96% between the 66 genes predicted in Fig. 5, so to avoid duplication, the functional enrichment analysis was not performed again. Instead, the scope of gene screening was narrowed, and redundant and interference genes were eliminated. Cytoscape was employed for building a possible co-expressed regulatory network, revealing that in these co-expressed genes, there might be a small regulatory network only covering 4 genes (hsa-mir-3923, AC117402.1, AC009646.2 and OPRK1) (Table 5).

### Gene expression and Co-expressed genome maps

**Figure 11:**
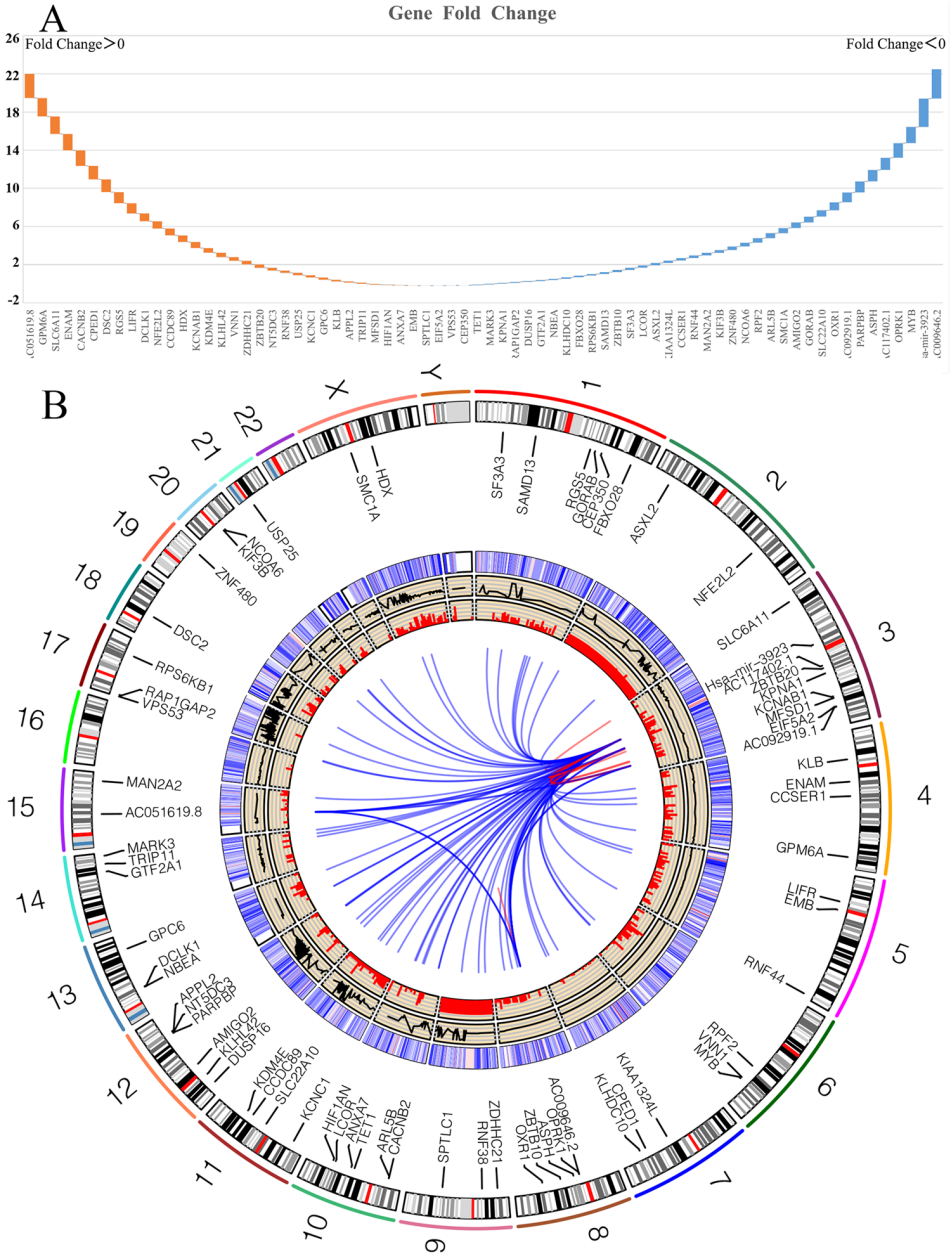
Circos plot derived from VENN and UpSet data. In order to explore the most represented reference fragments, all GRCh38.95 loci present in the genes co-expressed maps were deduced and merged. (**A**) 70 reference donor fragments were created, settled in the outermost track. Subsequently, the numbers of genes co-expressed maps containing each of these fragments’ labels were marked in the second track, except for duplicate counts. 91 pairs of genes were found to be co-expressed. In the inner sector, these pairs of genes sets co-expressed with lines were linked. The sum of genes co-expressed map alignments across the whole genome acted as the links for these genes co-expressed maps in the Circos plot. (**B**).

69 genes probably associated with hsa-mir-3923 were integrated and considered with the use of two different estimation methods. In addition, their variations in gastric cancer expression are shown in Fig. 11A. In accordance with the alinements of genes co-expressed maps to GRCh38.95, genomic regions for reference were identified promoting 70 genome sets’ composition.(hsa-mir-3923 marked as red dot) The genes co-expressed result and predictive analysis results in GEO (GSE13195 & GSE30727) and TCGA-STAD with hsa-mir-3923 were comprehensively analyzed based on the co-expression genes and miRNA predictive analysis results. To explore the most typical fragments for reference, all GRCh38.95 loci presenting in the genes co-expression maps were derived and combined. Subsequently, 70 donor fragments for reference were created and set in the outermost track. Next, the amounts of genes co-expressed maps covering respective label of these fragments were labelled in the second track, except for duplicate counts. 91 pairs of genes were found to display co-expression. In the internal sector, the mentioned pairs of genes sets co-expressed with lines were linked. The sum of genes co-expressed map alignments across the entire genome was the connections of these genes co-expressed maps in the Circos plot. (Fig. 11B)

## Discussion

It is reported that as a gene group, MicroRNA presented high or low expression in cancer. Besides, as oncogene, miRNAs can interact with mRNAs or lncRNAs, thereby modulating cancer development and accordingly regulating the cytological behavior^37,38^. The study found the large significance of miRNA hsa-mir-3923 in gastric carcinoma, and as a biomarker, miRNA hsa-mir-3923 could be applied to detecting the prognosis of GC. Analysis on the expression of hsa-mir-3923 in patients suffering GC helped to find factors which caused the high expression of hsa-mir-3923, i.e. the survival time, the histologic grade, the vital status, T stage, N stage, etc.

Thus far, rare studies represent the significance of hsa-mir-3923 located at chromosome 3p12.3^7,8^. Though no publications have been available in STAD by far, hsa-mir-3923 was reported to be significantly up-regulated in clinical STAD tissues with normal tissues. According to the study here, hsa-mir-3923 displayed high expression in gastric carcinoma, which is consistent with other studies about tumor. Note that hsa-mir-3923 expression was significantly elevated from T1 to T4, histologic grade varied from G1 to G3 and clinical stage varied from stage I to stage IV, suggesting its relevance to the progression of cancer. Besides, the hsa-mir-3923 expression was higher in patients survival time≤3 years than survival timeå 3 years, revealing its relevance to cancer prognosis and the necessity for subgroup study. Moreover, hsa-mir-3923 was more highly expressed in the deceased as compared with the living, so it is necessary to explore its link with the survival. After the analysis of the M & N stage of GC, though the statistical results were not significant, the expression level of hsa-mir-3923 in M1 & N1 phase was higher than that in M0 & N0 phase. To exclude tissue-specific interference, gene expression data were collected for various cancer types recorded in the TCGA database. As revealed in lateral comparison, this special miRNA is highly expressed among common tumor types.

Some existing studies also explored the way hsa-mir-3923 affects the occurrence and development of tumor^7,39^. According to large-scale clinical statistics, its obvious high-expression phenomenon was identified in the development of some tumor cell lines^8,40^. In the present study, hsa-mir-3923 is capable of affecting the initiation and proliferation of tumor, explaining that it is clinically associated with the TNM classification. Hsa-mir-3923 is tightly associated with cancer prognosis. In the meantime, it was found that the higher the hsa-mir-3923 expression, the poorer the OS will be, particularly in age (age ≤ 55), gender (female), advanced T stage (T3/4), M1 stage, advanced pathological stage (G3/4) and advanced clinical stage (stage 3/4). The independent prognostic effect of hsa-mir-3923 on the OS of patients was revealed from the results of Cox analysis; therefore, it could monitor the GC as a biomarker. Some studies have also suggested that besides affecting some of the common biological functions of tumor cells, the miRNA hsa-mir-3923 could also affect some specific cytological behaviors. After functional enrichment analysis of hsa-mir-3923, it was reported that this special miRNA was tightly related to NLS (Nuclear localization sequence) bearing protein import into nucleus, piRNA metabolic process, regulation of alternative mRNA splicing by spliceosome, pre mRNA binding and enhancement of RNA polymerase activity.

To delve into the biological role of miRNA hsa-mir-3923 in GC, we comparatively analyzed several miRNA databases (e.g., miRDB, miRPathDB, TargetScan, miRNAWalk, and miRTarBase) to identify 66 genes that might be tightly related to hsa-mir-3923. First, as suggested from the functional protein association network, several small regulatory networks might exist in the whole system, also indicating a probable hierarchy of regulatory networks involved in hsa-mir-3923. After functional enrichment of these genes with GO and KEGG, these genes were reported to be critical to the following biological behaviors. First, this hsa-mir-3923 could regulate the activity of many dioxygenases (e.g., 2,4-dichlorophenoxyacetate alpha-ketoglutarate dioxygenase activity, and hypophosphite dioxygenase activity). Many of the mentioned enzyme molecules were involved in the oxidative demethylation of biological molecules. Under normal conditions, it acted as one of the ways to maintain normal methylation/demethylation^41^. If under overly high activity of these enzymes, the degree of histone demethylation in some segments of the nucleus of tumor cells could decrease, making the chromosome structure more relaxed and facilitating gene transcription^42,43^. With the enhancement of these enzyme activities, it can also cause the maintenance of DNA demethylation of chromosomal sites containing CpG islands. The above biological behavior of has-mir-3923 can directly or indirectly promote the transcriptional activity of oncogenes by affecting the chromosomal structure, DNA stability, the binding capacity of transcription factors, etc. Besides, according to the results of functional enrichment analysis, the molecule could also participate in the oxidation of lipids, expedite the metabolism of lipids to reduce the damage of ROS to tumor cells and reduce the autophagy and apoptosis attributed to ROS^44^. Furthermore, such oxidation process could also facilitate the metabolism of considerable metabolic products (e.g., pantothenic acid and acetyl-CoA) into the mitochondria and participate in the Warburg Effect to promote the energy supply of tumor cells^45^. This molecule could also affect energy-related pathways (e.g., the Pi3k-MAPK signaling pathway) to regulate the cAMP concentration and calcium ion concentration in cells, which may promote the epithelial-mesenchymal transition (EMT) process in cells to induce tumor metastasis^46^.

According to the analysis on TCGA, we analyzed the data from GEO database regarding the co-expression. Then a comprehensive analysis was carried out on the co-expression result of genes in GEO and that in TCGA with hsa-mir-3923. Based on the result of WGCNA in R, lncRNA, miRNA and mRNA are associated with each other (Power Value=0.85), and they are specific genes which are related to the co-expression grid about hsa-mir-3923. A regulatory network with possible co-expression showed that these genes which are associated with each other regarding the co-expression have a small regulating network which contains four genes. Review of many miRNA databases helped to get 66 target genes with larger relevance in the gastric cancer and these genes could be found in large regulating network. Integration of data from GEO, TCGA and miRNA databases helped to get 69 genes which exhibit a close association with different types of has-mir-3923 such as lncRNAs and mRNAs. At last, we combined these data with the expression of gene to predict possible regulating networks. GRCh38.95 was used to carry out whole-genome mapping, which covers the abovementioned 70 genes that represent regions with the largest expression difference in cancer genome. On that account, it is available to directly observe the co-expressed regulating network without needing to complexly calculating the complicated assumptions.

To the best of our knowledge, the present study proved the great effect of miRNA hsa-mir-3923 on the prognosis of GC for the first time. The study together with other related studies was also beneficial for finding that hsa-mir-3923 is very important in GC. However, future studies are supposed to verify these findings relying on clinical trials, as an attempt to ensure the wide application of hsa-mir-3923 for the prognosis of GC.

## Conclusion

Our study reported that the high-expression of hsa-mir-3923 was noticeably up-regulated in GC patients and associated with several clinical features and undesirable prognosis, so miRNA hsa-mir-3923 could act as an effective biomarker for the prognosis of gastric carcinoma patients.

## Data Availability

Availability of data and materials The Cancer Genome Atlas-Stomach Adenocarcinoma (TCGA-STAD) and Gene Expression Omnibus (GEO) (GSE13195 & GSE30727). The data used in this article was downloaded in June 2019.
